# Restoring circadian disrupted gut microbial metabolite rhythms with phytochemicals: a new avenue against metabolic disease

**DOI:** 10.3389/fmicb.2026.1801367

**Published:** 2026-05-08

**Authors:** Lipeng Wu, Shaozhen Li, Fangkai Han, Jun Guo, Xingtao Zhang, Lisheng Xu

**Affiliations:** 1 College of Biology and Food Engineering, Suzhou University, Suzhou, Anhui, China; 2 Collaborative Technology Service Center for the High-Value Processing of Green Agricultural Products (Prepared Foods) in the Yangtze River Delta Region, Suzhou, Anhui, China

**Keywords:** circadian rhythm, gut microbiota, metabolic syndrome, microbial metabolites, phytochemicals

## Abstract

The global epidemic of metabolic diseases—encompassing obesity, type 2 diabetes mellitus (T2DM), non-alcoholic fatty liver disease (NAFLD), and cardiovascular disease—represents a defining public health challenge of our era. The traditional model of simple caloric excess vs. expenditure has proven insufficient, giving way to a paradigm that acknowledges complex interactions between genetics, environment, and lifestyle, mediated by intricate physiological systems. Among these, the host circadian timing system and the gut microbiome have ascended as pivotal, deeply intertwined regulators of metabolic homeostasis. The gut microbiome, far from a static collection of microbes, constitutes a dynamic and metabolically active community whose composition and, critically, its functional output exhibit profound and predictable diurnal oscillations. The host's circadian clocks regulate microbial rhythms primarily by controlling the daily cycle of feeding and fasting. Modern life induces circadian disruption (CD) through ubiquitous exposure to artificial light at night, shift work, social jet lag, and erratic eating patterns. It perturbs the rhythmic dynamics of the gut ecosystem, leading to a fundamental dysregulation in the temporal production of key microbial metabolites. These metabolites, including short-chain fatty acids, secondary bile acids (BAs), indoles and other tryptophan derivatives, function as indispensable chemical messengers that coordinate peripheral metabolism, immune responses, and energy homeostasis in a precise, time-of-day-dependent manner. Their desynchronization—manifesting as mistimed, deficient, or incessant signaling—directly instigates the core pathologies of metabolic disease: insulin resistance, adipocyte dysfunction, hepatic lipid accumulation, and chronic low-grade inflammation. This review synthesizes current evidence to delineate the multilevel mechanisms through which CD drives the dysregulation of gut microbiome metabolite rhythms and establishes the causal pathways linking this dysrhythmia to metabolic pathogenesis. Furthermore, we undertake a critical evaluation of the promising therapeutic potential of dietary phytochemicals—a diverse class encompassing polyphenols, glucosinolates, and prebiotic fibers—to act as chrono-therapeutic agents. Through their multifaceted capacity to remodel microbial ecology, calibrate microbial enzymatic output, and reinforce host circadian-metabolic coupling, phytochemicals present a novel, physiologically aligned, and sustainable dietary strategy for the prevention and management of metabolic disorders. We conclude by outlining key translational challenges and propose future research directions essential for harnessing the potential of the “clock-microbiome-metabolite” axis within the framework of precision nutrition and medicine.

## 1 Introduction

Metabolic diseases are staggeringly prevalent worldwide, creating an unsustainable healthcare burden. Their etiology is complex, moving beyond simplistic models of energy balance (Zheng et al., 2018). This complexity has driven the search for new pathophysiological mechanisms, focusing research on two deeply interconnected regulatory systems: the host's circadian clock and the gut microbiome (Thaiss et al., 2014). The mammalian circadian system is an evolutionarily conserved, hierarchical timekeeping network that enables organisms to anticipate and optimally adapt to the predictable 24-h oscillations in their environment (Henao-Mejia et al., 2012). A master pacemaker in the hypothalamic suprachiasmatic nucleus (SCN) orchestrates this system. Its key function is to synchronize subsidiary, cell-autonomous clocks found in virtually every peripheral tissue, such as the liver, adipose tissue, skeletal muscle, and the gastrointestinal tract (Tahara and Shibata, 2016). At the molecular core of these clocks lie interlocked transcription-translation feedback loops driven by core clock genes such as *Clock, Bmal1, Period (Per1/2)*, and *Cryptochrome (Cry1/2)* (Takahashi, 2017). These loops govern the rhythmic expression of a significant portion (estimates range from 10 to 50% depending on the tissue) of the mammalian transcriptome, thereby imposing temporal order on a vast array of physiological processes (Segers and Depoortere, 2021). These include the sleep-wake cycle, core body temperature, and hormone secretion patterns, such as cortisol's morning peak and melatonin's nocturnal rise. Most critically for this discussion, it governs systemic metabolism—including glucose, lipids, cholesterol, and energy expenditure—on a circadian schedule (Takahashi, 2017). The maintenance of synchrony between these internal oscillators and external zeitgebers (“time-givers”), primarily the light-dark cycle and the timing of food intake, is a non-negotiable prerequisite for metabolic health. On the other hand, misalignment, or CD, is a potent stressor metabolic dysfunction such as diabetes and obesity (Karlsson et al., 2013; Liu et al., 2017).

Parallel to this temporal regulatory framework exists the gut microbiome, now recognized not as a passive bystander but as a virtual, acquired endocrine organ of immense metabolic capacity and influence (Rastelli et al., 2019; Schroeder and Bäckhed, 2016). This complex consortium of trillions of bacteria, archaea, viruses, and fungi harbors a collective genome (the microbiome) encoding millions of genes, dwarfing the human genome in its functional potential (Qin et al., 2010). Endowed with this genetic arsenal, the microbiome carries out vital nutritional roles: it breaks down indigestible fibers and polyphenols, and synthesizes essential vitamins like vitamin K and B vitamins. Beyond nutrition, it also educates the host immune system and provides resistance against pathogenic colonization (Rodriguez et al., 2015; Selma et al., 2009). A paramount function is the biotransformation of dietary and host-derived precursors into a vast repertoire of bioactive metabolites (Fabbiano et al., 2018). These microbial products include short-chain fatty acids, secondary bile acids (BAs), and various tryptophan catabolites and so on (Janssens et al., 2018). They serve as key signaling molecules that influence host physiology, both locally within the gut and systemically, reaching and affecting distant organs like the liver, brain, and adipose tissue (Krautkramer et al., 2016). A paradigm-shifting discovery in the last decade has been the revelation that the gut ecosystem is not in a steady state. Its taxonomic composition, functional gene expression, and consequent metabolic output exhibit robust, predictable diurnal oscillations (Liang et al., 2015; Thaiss et al., 2014). This microbial rhythmicity is not autonomous but is entrained by host-derived signals, with feeding time being the dominant cue (Stokkan et al., 2001). Consequently, the intestinal concentrations of key microbial metabolites undergo a daily ebb and flow. These fluctuating metabolites act as timing signals, or “microbial zeitgebers,” for host peripheral tissues. They fine-tune metabolic pathways to anticipate feeding cycles, thereby optimizing energy harvest, storage, and utilization (Leone et al., 2015).

The modern industrialized environment is replete with factors that induce chronic CD. These factors include pervasive exposure to artificial light at night, the demands of shift work and frequent transmeridian travel, and the cultural normalization of erratic eating patterns. Such eating often extends caloric intake deep into the biological night. Together, they create a state of chronic CD (Scheer et al., 2009). Epidemiological and experimental data robustly link CD to increased risk for obesity, T2DM, NAFLD, and cardiovascular disease. A critical mechanistic bridge connecting CD to metabolic dysfunction is its profound and disruptive impact on the gut microbiome (Aoki et al., 2021; Knutson and Van Cauter, 2008). CD flattens the normal, health-associated diurnal oscillations of the microbiota, precipitating a state of dysbiosis. This dysbiosis is defined not merely by a static change in which species are present, but by a more profound loss of functional rhythmicity—a breakdown in the temporal organization of microbial community activities (Voigt et al., 2014). This arrhythmia leads to the desynchronized, mistimed, or diminished production of key microbial metabolites. Consequently, the crucial, time-sensitive dialogue between the microbiome and host metabolic organs (liver, adipose, muscle, and pancreas) becomes garbled. The loss of rhythmic metabolite signaling has several detrimental effects. It impairs metabolic flexibility (the ability to efficiently switch between fuel sources), predisposes to ectopic lipid deposition, dysregulates glucose production, and fuels a state of chronic and low-grade inflammation. Together, these effects establish a permissive environment for metabolic diseases (Kaczmarek et al., 2017; Paschos and FitzGerald, 2017). In this context, dietary phytochemicals—the diverse array of bioactive, non-nutrient compounds produced by plants—emerge as a compelling and natural therapeutic opportunity (Beam et al., 2021; Hur and Lee, 2015). Compounds such as the polyphenol families (e.g., flavonols like quercetin, stilbenes like resveratrol, curcuminoids like curcumin), glucosinolates (e.g., sulforaphane from cruciferous vegetables), and the broad category of dietary fibers have demonstrated a remarkable spectrum of biological activities (Feng et al., 2019). These include prebiotic effects that selectively nourish beneficial bacteria, direct antimicrobial actions against potential pathobionts, and the ability to modulate host signaling pathways involved in inflammation, oxidative stress, and metabolism (Cardona et al., 2013; Feng et al., 2019). They possess an inherent capacity to interface with both the microbial community and host physiology. This uniquely positions them as strategic agents. As such, they are capable of restoring the integrity of the disrupted “clock-microbiome-metabolite” axis (Sommer and Bäckhed, 2013). By acting as microecological engineers, metabolic pathway modulators, and host system stabilizers, phytochemicals offer a multi-targeted, dietary approach to chronotherapy (Han et al., 2021).

**Figure 1:**
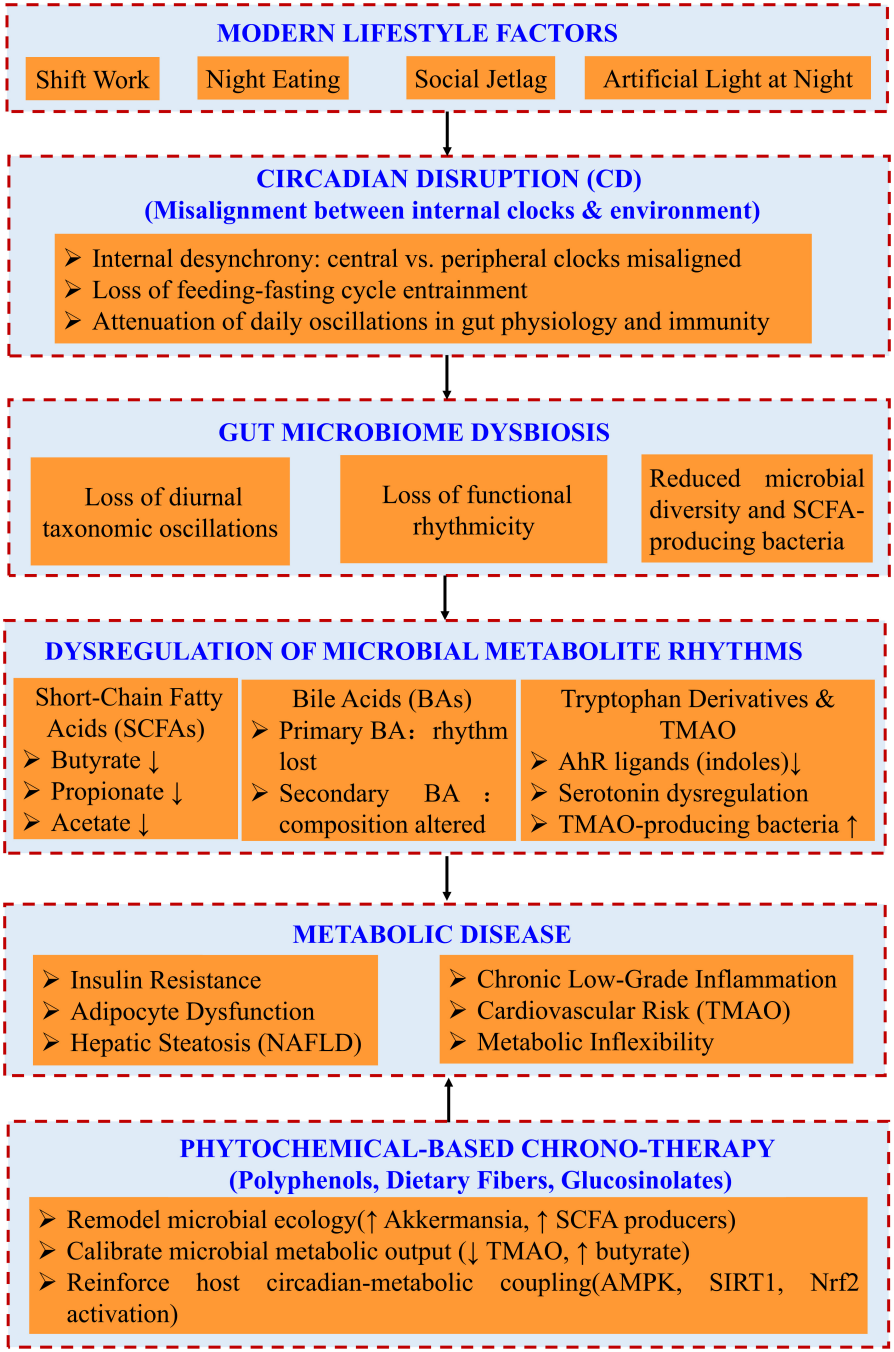
Overview of the pathogenic axis linking circadian disruption to metabolic disease via gut microbiome dysregulation and the therapeutic role of phytochemicals. (Modern lifestyle factors (shift work, night eating, and social jetlag) induce circadian disruption, characterized by internal desynchrony and flattened microbial rhythms. This perturbs the diurnal oscillations of the gut microbiome, leading to dysregulated production of key microbial metabolites (SCFAs, bile acids, tryptophan derivatives), which drive the core pathologies of metabolic syndrome. Dietary phytochemicals counteract this axis by remodeling microbial ecology, calibrating metabolite output, and reinforcing host circadian-metabolic coupling).

Therefore, the primary objectives of this review are fourfold: (1) to provide a detailed, mechanistic exposition of how host circadian rhythms regulate the gut microbiome and how various forms of CD dismantle this regulation, leading to microbial and metabolite arrhythmia; (2) to elucidate the specific pathophysiological consequences of disrupted rhythms in key microbial metabolites (SCFAs, BAs, tryptophan derivatives) for the development and progression of metabolic disease endpoints; (3) to comprehensively evaluate the preclinical and clinical evidence supporting the role of dietary phytochemicals as chrono-therapeutics capable of recalibrating this axis at multiple levels; and (4) to discuss the significant translational challenges, future research trajectories, and the promise of personalized chrono-nutrition as a framework for combating metabolic disease. The conceptual framework of this review, illustrating the sequential cascade from circadian disruption to metabolic disease and the therapeutic intervention points of phytochemicals, is summarized in Figure 1.

## 2 Impact of circadian disruption on the gut microbiome and its metabolic rhythms

### 2.1 Host circadian regulation of the microbiome: a multilayered dialogue

The host exerts sophisticated, time-gated control over its gut microbial inhabitants through a combination of direct genetic regulation of the intestinal environment and indirect behavioral and systemic hormonal pathways. Together, these forces create an oscillating niche whose hospitality and selective pressure vary over the 24-h cycle (Spor et al., 2011; Sgritta et al., 2019).

#### 2.1.1 Direct circadian control of the gastrointestinal niche

Peripheral circadian clocks within intestinal epithelial cells (enterocytes, colonocytes), specialized secretory cells (Paneth cells, goblet cells), and associated immune cells (e.g., dendritic cells, macrophages) directly regulate the local luminal and mucosal environment. The molecular clockwork within these cells controls the rhythmic expression of proteins critical for gut structure, function, and defense. A prime example is the clock-controlled production of antimicrobial peptides (AMPs), such as regenerating islet-derived protein 3γ (Reg3γ) and α-defensins, by Paneth cells in the small intestine. These AMPs are secreted into the gut lumen in a diurnal pattern, creating a temporal landscape of antimicrobial pressure that profoundly shapes microbial community structure by selectively inhibiting or eliminating certain bacterial taxa while allowing others to flourish during specific windows (Niu et al., 2024; Mukherji et al., 2013). Furthermore, the integrity of the intestinal epithelial barrier, maintained by dynamic protein complexes like tight junctions (e.g., occludin, claudins) and adherens junctions, also exhibits circadian regulation. The rhythmic expression and phosphorylation of these junctional proteins dictate the paracellular permeability of the gut lining, which in turn affects the degree of microbial-associated molecular pattern translocation and the nature of host-microbe interaction at the mucosal surface (Fujisaka et al., 2018). As demonstrated in intestinal epithelium-specific *Bmal1* knockout mice, disruption of these local intestinal clocks led to a loss of microbial diurnal fluctuations and compositional dysbiosis even under strictly controlled feeding conditions. This provided definitive evidence for a direct, feeding-independent circadian influence on the microbiome orchestrated from within the gut tissue itself (Liang et al., 2015).

#### 2.1.2 Indirect regulation via systemic zeitgebers and host rhythms

While local clocks play a fundamental role, the most potent external signal for entraining microbial rhythms is the host's behavioral cycle of feeding and fasting (Patterson and Sears, 2017). Time-restricted feeding (TRF), which consolidates all caloric intake to a consistent 8–12 h window aligned with the active phase, has been shown to be a powerful driver and restorer of microbial oscillations (Mattson et al., 2014). Notably, TRF can rescue microbial rhythmicity and improve metabolic parameters even in mice with genetically or surgically ablated central (SCN) clocks. It was demonstrated that feeding time itself was a primary, non-photic regulator for the gut microbiome (Sharon et al., 2016; Zarrinpar et al., 2014). This powerful effect is mediated through two main channels: the rhythmic delivery of dietary substrates (the microbes' primary energy source) and the concomitant fluctuation of host-derived systemic signals that are themselves synchronized to the feeding cycle (Landgraf et al., 2015). These systemic signals include hormonal fluctuations, body temperature, and bile acid flux. The circadian secretion of glucocorticoids (cortisol in humans, corticosterone in rodents), which peak around the wake-time, has broad immunomodulatory and metabolic effects (Delezie and Challet, 2011). Glucocorticoid receptors are present in some bacteria, and these hormones can influence microbial gene expression and growth dynamics (Ma et al., 2017). Similarly, the rhythmic patterns of appetite-regulating hormones like leptin (satiety signal) and ghrelin (hunger signal) may have direct or indirect effects on microbial physiology, potentially through changes in gut motility or nutrient sensing (Paulose et al., 2016). In addition, the robust circadian oscillation in core body temperature (higher during active phase, lower during rest) creates a thermally fluctuating environment within the gut lumen. This daily temperature cycle can selectively influence the growth rates and metabolic activities of different microbial species, as bacterial enzymes have optimal temperature ranges (Qin et al., 2012; Ríos-Covián et al., 2016). Besides, the liver clock tightly regulates the synthesis of primary BAs via transcriptional control of the rate-limiting enzyme cholesterol 7α-hydroxylase (CYP7A1). This results in a robust diurnal rhythm in the hepatic synthesis and subsequent biliary secretion of primary BAs into the duodenum (Govindarajan et al., 2016). BAs are potent detergents with inherent antimicrobial properties. Their rhythmic flux into the intestine imposes a significant, time-varying chemical and selective pressure on the microbial community, favoring bacteria equipped with bile acid resistance and transformation enzymes over those sensitive to BA detergent action (Turnbaugh et al., 2006).

### 2.2 Major environmental desynchronizing factors in modern life

Contemporary society is characterized by several ubiquitous behaviors and occupational demands, acting as chronic circadian stressors. They effectively decouple our internal biological clocks from natural environmental cycles and, by extension, from the rhythmic life of the gut microbial community (Smits et al., 2017; Vrieze et al., 2012).

#### 2.2.1 Irregular and night-time eating

The erosion of structured meal times and the trend of consuming significant calories in the late evening or biological night are now prevalent. These habits delivers nutrient substrates to the gut ecosystem at an aberrant circadian phase—a period normally associated with fasting, sleep, and insulin resistance. This disrupts the coordinated, anticipatory metabolic response of the microbiota. Some rodent studies provided incontrovertible evidence: compared with mice fed with isocaloric high-fat diets during their active (dark) phase, mice consuming the same diet exclusively during their normal resting (light) phase developed significantly greater weight gain, adiposity, glucose intolerance, hyperinsulinemia, and hepatic steatosis (Arble et al., 2009; Bisanz et al., 2019; Shimizu et al., 2018). Controlled human intervention studies also mirrored these findings. Late-evening meals or shifting food intake to later hours impaired postprandial glucose tolerance, altered lipid metabolism (e.g., leading to higher postprandial triglycerides). Additionally, it reduced diet-induced thermogenesis compared to eating earlier in the day (Wehrens et al., 2017). Metagenomic and metabolomic analyses revealed that such mistimed eating not only altered the absolute abundance of key bacterial families (e.g., reducing *Lachnospiraceae*) but, more importantly, dampened the normal rhythmicity of microbial taxonomic and functional gene profiles involved in carbohydrate and amino acid metabolism (Zarrinpar et al., 2014).

#### 2.2.2 Shift work and transmeridian travel

Shift work and transmeridian travel can immediately disrupt the body's internal timing. This disruption often becomes persistent, causing the master light-sensitive clock to fall out of sync with clocks in metabolic organs. Specifically, the central SCN clock—entrained by light—becomes misaligned with peripheral clocks, which are more strongly set by the altered timing of food intake, sleep, and activity (Tu et al., 2021). Epidemiological data consistently showed that shift workers, particularly those on rotating night shifts, had a 20%−40% increased risk of developing obesity, T2DM, and coronary heart disease, even after adjusting for lifestyle factors (Wang X. S. et al., 2011). Experimental models, both in human volunteers under controlled laboratory conditions simulating night-shift work and in mice subjected to weekly phase-advancements mimicking jet lag, demonstrated that circadian misalignment rapidly and reproducibly leads to significant alterations in gut microbiota composition. These changes are typically characterized by a decrease in the relative abundance of beneficial, SCFA-producing bacteria from families such as *Lachnospiraceae* and *Ruminococcaceae*, an increase in taxa associated with inflammation (e.g., *Coprococcus*), and a profound dampening or complete obliteration of diurnal microbial fluctuations (Voigt et al., 2014; Kervezee et al., 2020). The microbial community loses its temporal structure, becoming static in a dysbiotic state.

#### 2.2.3 Chronic social jet lag

Social jet lag is a highly prevalent condition of chronic mismatch. It occurs between an individual's endogenous circadian preference (chronotype, such as “night owl” or “morning lark”) and their socially imposed sleep-wake schedule, like early work or school start times. This mismatch results in recurrent, weekly bouts of circadian misalignment, typically on work or school days (Wang X. S. et al., 2011). Even mild social jet lag (a discrepancy of ≥2 h) is associated with adverse metabolic profiles, including higher body mass index (BMI), increased insulin resistance (HOMA-IR), and unfavorable lipid markers (Roenneberg et al., 2012). Emerging evidence links social jet lag to distinct and measurable microbial signatures. These include an overall reduction in microbial alpha-diversity, a key marker of ecosystem health and stability. They also involved an enrichment of bacterial groups associated with inflammatory markers, suggesting this common, chronic CD exerts a selective pressure that reshapes the gut into a less favorable, less rhythmic state.

### 2.3 Manifestations and consequences of microbial rhythm dysregulation

Whether induced by irregular eating, shift work, or social jet lag, the common endpoint of these CD models is a functionally desynchronized gut ecosystem. The ultimate consequence of CD is a gut microbiome that is not only compositionally altered in a static sense but, more critically, is functionally desynchronized. This loss of temporal order and predictive dynamics has profound implications for host-microbe mutualism (Yamajuku et al., 2012).

#### 2.3.1 Attenuation of diurnal taxonomic and functional fluctuations

In a healthy, synchronized state, the relative abundances of specific bacterial taxa oscillate predictably over the 24-h period. For instance, members of the *Firmicutes phylum*, particularly those equipped with enzymes to break down complex dietary fibers, often peak in abundance during or shortly after the host's feeding phase (Nie et al., 2017; Oosterman et al., 2015). Conversely, mucin-degrading specialists like *Akkermansia muciniphila* (*Verrucomicrobia phylum*) might exhibit higher relative abundance during the fasting phase, feeding on host-derived mucosal glycans. CD, whether induced by genetic knockout of central clock genes (*Bmal1, Per1/2*) or by environmental manipulation like experimental jet lag, dramatically flattens these population oscillations (Thaiss et al., 2014). As a consequence, the microbial community loses its dynamic ebb and flow, becoming “stuck” in a compositionally and functionally monotonous state that is maladapted to the host's cyclically changing metabolic needs (Yang et al., 2022).

#### 2.3.2 Loss of transcriptional and metabolic pathway rhythmicity

In addition to shifts in which species are present, CD also disrupts the rhythmic expression of microbial genes, which is the primary driver of the community's function. Metatranscriptomic analyses (sequencing of microbial community RNA) revealed that under conditions of CD, the normal daily expression patterns of genes responsible for critical bacterial functions were severely blunted or lost (Yang et al., 2020). These include genes encoding carbohydrate-active enzymes (CAZymes) for dietary fiber breakdown, key enzymes in SCFA synthesis pathways (e.g., butyryl-CoA:acetate CoA-transferase for butyrate production), genes for flagellar assembly and bacterial motility, and transporters for nutrients like sugars and amino acids (Kumar et al., 2020; Wang Z. et al., 2011). This transcriptional arrhythmia translates directly into a mistimed production of metabolites. Instead of a coordinated, anticipatory pulse of SCFAs or a rhythmic conversion of primary to secondary BAs aligned with host metabolic demands, microbial metabolic output became erratic, constitutively subdued, or inappropriately persistent. The normal signal becomes noise (Mukhopadhya and Louis, 2025; Yoo et al., 2004).

#### 2.3.3 Causal evidence from fecal microbiota transplantation (FMT)

The most compelling evidence that a CD-perturbed microbiome directly drives metabolic dysfunction comes from FMT experiments. They showed that a circadian-disrupted microbiome was a direct and sufficient driver of metabolic dysfunction, not just a passive correlate. Thus, FMT studies provided definitive proof of causality (Zarrinpar et al., 2016). These studies involved transplanting the gut microbiota from donor mice subjected to chronic jet lag or from circadian clock mutant mice (e.g., *Per1/2* double knockout) into germ-free (axenic) or antibiotic-treated wild-type recipient mice. Remarkably, recipients of the “CD-microbiota” developed significant glucose intolerance, increased adiposity, and other metabolic disturbances, despite being housed under normal, regular light-dark cycles and having no inherent circadian defect themselves (Voigt et al., 2014; Leone et al., 2015). This demonstrated conclusively that the dysregulated, arrhythmic microbial community itself is a transmissible pathogenic entity, capable of inducing metabolic disease in a previously healthy host (Zhang et al., 2017). It solidified the gut microbiome as a key mediator in the causal pathway from CD to metabolic syndrome (Zhang et al., 2021).

Table 1 summarizes the key impacts of different CD models on the gut microbiota and the consequent host metabolic phenotypes, providing a concise overview of the experimental evidence discussed in this section.

**Table 1:** Impact of CD models on gut microbiota and metabolic phenotypes.

Disruption model	Key microbiota alterations	Metabolic phenotype in host	References
Genetic (e.g., *Bmal1* KO, *Clock* mutant)	Loss of diurnal oscillations; Reduced *Lactobacillaceae*; Increased Firmicutes/Bacteroidetes ratio	Glucose intolerance, obesity, hepatic steatosis, hyperphagia	Liang et al. (2015); Mukherji et al. (2013); Zuo et al. (2019)
Environmental (Jet lag/Shift work)	Damped rhythmicity; Decreased alpha-diversity; Enrichment of pro-inflammatory taxa (e.g., *Coprococcus*)	Weight gain, insulin resistance, increased systemic inflammation, hypertriglyceridemia, impaired glucose tolerance	Voigt et al. (2014); Kervezee et al. (2020); Blander et al. (2017); Van Hul and Cani (2023)
Diet-induced (night-time feeding)	Altered composition independent of diet quality; Reduced SCFA-producing bacteria (e.g., *Roseburia, Faecalibacterium*)	Hyperphagia, increased adiposity, disrupted lipid metabolism (hepatic steatosis), impaired glucose tolerance	Zarrinpar et al. (2014); Wehrens et al. (2017); Shimizu et al. (2018)
FMT from CD donor	Successful establishment of donor's dysbiotic/arrhythmic community in recipient gut	Transfer of donor's metabolic phenotype: glucose intolerance and increased adiposity in healthy recipient	Voigt et al. (2014); Leone et al. (2015)

## 3 Pathophysiological mechanisms of dysregulated microbial metabolite rhythms in metabolic disease

The loss of rhythmicity in microbial metabolite production is not only a benign or academic observation. It actively cut off critical communication lines between the gut and systemic metabolic organs. As a result, the mistimed, deficient, or excessive signaling of key microbial metabolites drives disease pathogenesis through some specific and well-defined molecular pathways (Cussotto et al., 2018; Zhou et al., 2018).

### 3.1 The collapse of short-chain fatty acid rhythms

SCFAs—primarily acetate (C2), propionate (C3), and butyrate (C4)—are the principal end-products of microbial fermentation of dietary fiber and resistant starch. Their production normally peaks several hours after a meal, aligning with the host's postprandial anabolic state. CD disrupts this rhythm, often leading to both an overall reduction in total SCFA levels and, more insidiously, an erasure of their temporal signature, resulting in a flat, non-rhythmic production profile (Tahara et al., 2018).

#### 3.1.1 Butyrate

Butyrate is like the chrono-metabolic gatekeeper and epithelial guardian. Butyrate is a multi-faceted signaling molecule with profound local and systemic effects (Zietak et al., 2016). Locally in the colon, it serves as the preferred energy source for colonocytes (intestinal epithelial cells). Its rhythmic supply regulates the circadian expression of genes involved in epithelial cell proliferation, differentiation, and, crucially, the assembly of tight junction proteins (e.g., occludin, claudins, zonula occludens-1). This maintains the diurnal integrity of the gut barrier (Fujisaka et al., 2018). A disrupted butyrate rhythm deranges the function of this barrier, leading to increased intestinal permeability or “leaky gut.” This allows the translocation of bacterial pro-inflammatory molecules, most notably lipopolysaccharide (LPS) from Gram-negative bacteria, into the portal and then systemic circulation—a condition termed metabolic endotoxemia (Steliou et al., 2012). Elevated circulating LPS triggers a chronic low-grade inflammatory state via activation of Toll-like receptor 4 (TLR4) on immune cells, a key driver of systemic insulin resistance and adipose tissue inflammation (Cani, 2018). In addition to its local guardianship of the epithelial barrier, butyrate exerts systemic epigenetic influence as a potent inhibitor of histone deacetylases (HDACs). Rhythmic HDAC inhibition in peripheral tissues like the liver is an important epigenetic mechanism contributing to the circadian expression of metabolic genes. For example, butyrate's rhythmic levels help fine-tune the liver's daily production of glucose. Its daily ebb and flow helps set the pace for the expression of phosphoenolpyruvate carboxykinase (PCK1), a rate-limiting enzyme in hepatic gluconeogenesis (Zubcevic et al., 2019). Loss of this timing signal can lead to inappropriately timed or excessive hepatic glucose output during periods when it should be suppressed (e.g., the fed state), directly contributing to fasting and postprandial hyperglycemia (Kasukawa et al., 2012; Santisteban et al., 2017). Butyrate also activates G-protein coupled receptors (GPCRs) like GPR109a on immune cells, promoting anti-inflammatory interleukin-10 (IL-10) production, and may entrain peripheral clocks, further linking microbial metabolism to host circadian physiology (Tahara et al., 2018).

#### 3.1.2 Propionate and acetate: mistimed appetite, gluconeogenic, and lipogenic signals

Propionate is largely cleared by the liver via first-pass metabolism, where it can serve as a substrate for gluconeogenesis. In the intestinal lumen, propionate activates GPCRs GPR41 and GPR43 on enteroendocrine L-cells. This GPR activation stimulates the rhythmic release of the anorexigenic (satiety) hormones peptide YY (PYY) and glucagon-like peptide-1 (GLP-1). PYY slows gastric emptying and intestinal transit (the “ileal brake”), while GLP-1 potentiates glucose-dependent insulin secretion, inhibits glucagon release, and promotes satiety in the brain (Chambers et al., 2015). Arrhythmic propionate production may blunt this crucial postprandial satiety and insulinotropic signal, potentially contributing to overeating and impaired glycemic control. Acetate, the most abundant SCFA in circulation, can reach the peripheral tissues. In the liver, it can serve as a substrate for *de novo* lipogenesis (DNL), the process of converting carbohydrates to fatty acids (Han et al., 2021). When the availability of acetate is mistimed—for example, peaking during a circadian phase associated with insulin resistance (as occurred in night-eating)—it may contribute to hepatic lipid accumulation and dyslipidemia (Perry et al., 2016). In addition, acetate can cross the blood-brain barrier, influence central appetite regulation and may have effects on hypothalamic function (Alenghat, 2015).

### 3.2 Temporal disarray in bile acid metabolism and signaling

The bile acid pool—its size, composition, and enterohepatic circulation—exhibits a robust diurnal rhythm. This rhythm is governed by the liver clock and meticulously modified by the gut microbiome. CD disrupts this rhythm at multiple and interconnected levels (Asher and Sassone-Corsi, 2015).

#### 3.2.1 Disruption of hepatic synthesis and microbial transformation rhythms

Hepatic expression of *Cyp7a1*, the gene encoding the rate-limiting enzyme in the classical pathway of bile acid synthesis, is under strong circadian control, typically peaking during the active phase (Govindarajan et al., 2016). This drives a rhythm in the secretion of primary BAs (cholic acid, chenodeoxycholic acid) into the bile. Upon reaching the distal ileum and colon, a subset of gut bacteria, notably within the Clostridium cluster (e.g., *Clostridium scindens*), perform a critical biotransformation: 7α-dehydroxylation, converting primary BAs into secondary BAs like deoxycholic acid (DCA) and lithocholic acid (LCA) (Sayin et al., 2013). CD alters both the host's synthetic rhythm (via clock disruption) and the abundance/activity of these specialized transforming bacteria, resulting in an aberrant bile acid pool. This pool is characterized by an altered ratio of primary to secondary BAs, a shift in hydrophobicity, and a loss of the normal daily variation in composition and concentration (Kuipers et al., 2014). The side effect is an arrhythmic delivery of BAs signals to intestinal and systemic receptors (Beli et al., 2018).

#### 3.2.2 Pathophysiological consequences of uncoordinated FXR and TGR5 activation

BAs are endogenous ligands for two key signaling receptors: the farnesoid X receptor (FXR), a nuclear receptor, and Takeda G protein-coupled receptor 5 (TGR5/GPBAR1). Their rhythmic activation is essential for metabolic coordination (Bishehsari et al., 2015).

FXR Signaling: in the ileal enterocyte, rhythmic activation of FXR by BAs induces the expression and secretion of fibroblast growth factor 19 (FGF19; FGF15 in mice). FGF19 travels via portal blood to the liver, where it binds its receptor (FGFR4/β-KLOTHO complex) to suppress CYP7A1 expression, completing a negative feedback loop that rhythmically controls BAs synthesis. FGF19 also regulates hepatic glycogen and protein synthesis. In the liver itself, FXR activation regulates the expression of genes involved in gluconeogenesis (suppressing *Pck1* and *G6pc*), lipogenesis (inhibiting SREBP-1c), and VLDL-triglyceride secretion (Kuipers et al., 2014; Wahlström et al., 2016).

TGR5 Signaling: activation of TGR5 on intestinal L-cells stimulates GLP-1 secretion, enhancing insulin secretion and promoting satiety. In brown adipose tissue and skeletal muscle, TGR5 activation increases energy expenditure and thermogenesis via a cyclic AMP (cAMP)-dependent mechanism that upregulates type 2 iodothyronine deiodinase (DIO2), converting thyroxine (T4) to active triiodothyronine (T3) (Canfora et al., 2015; Donohoe et al., 2011).

CD leads to arrhythmic tissue exposure to BAs. This results in the uncoordinated, suboptimal, or constitutive activation of FXR and TGR5 receptors. Such dysregulation disrupts the normal phasing of metabolic processes. For instance, postprandial suppression of hepatic glucose production may fail, lipogenesis may stay chronically high, the BAs feedback may become dysregulated (increasing cholestatic risk), and thermogenesis may be blunted. Collectively, these disturbances promote hyperglycemia, dyslipidemia, and weight gain—the hallmarks of metabolic disease.

### 3.3 Role of other rhythmic microbial signaling molecules

#### 3.3.1 Tryptophan metabolites: AhR ligands and serotonin precursors

Dietary tryptophan is metabolized through several competing pathways between host and microbe. Gut bacteria convert tryptophan into a variety of indole and derivative compounds, many of which are potent ligands for the aryl hydrocarbon receptor (AhR), such as indole, indole-3-aldehyde, indole-3-acetic acid, and indole-3-propionic acid (IPA) (Cheng et al., 2020). The activation of AhR in intestinal immune cells (e.g., innate lymphoid cells type 3, ILC3s) and epithelial cells is crucial for maintaining mucosal immune homeostasis, barrier function, and the production of the cytokine interleukin-22 (IL-22). IL-22, in turn, stimulates Paneth and goblet cells to produce antimicrobial peptides and mucins, reinforcing the barrier (Natividad et al., 2018). Microbial tryptophan metabolism also exhibits diurnal patterns tied to nutrient availability. Its disruption under CD may lead to a deficit in these beneficial AhR ligands, weakening the gut barrier, compromising mucosal immunity, and exacerbating local and systemic inflammation (Cho et al., 2019). Furthermore, the host's synthesis of the neurotransmitter serotonin, which regulates gut motility, mood, and platelet function, depends on the availability of its precursor, tryptophan. The microbiome competes for and metabolizes tryptophan, thereby influencing the host's serotonergic system. Disruption of this balance may link gut dysbiosis to neurobehavioral comorbidities which were commonly found in metabolic disease, such as depression and anxiety (Yano et al., 2015).

#### 3.3.2 Trimethylamine N-Oxide (TMAO): a CD-exacerbated cardiovascular risk factor

While TMAO levels in plasma may not exhibit a strong endogenous circadian rhythm in humans, the microbial production of its precursor, trimethylamine (TMA), is directly influenced by the availability of substrate from the diet (Cresci and Bawden, 2015). TMA is generated by specific gut bacterial enzymes (e.g., CutC/D, CntA/B) from dietary nutrients abundant in red meat, eggs, and certain fish, namely, choline, L-carnitine, and phosphatidylcholine. CD, by altering feeding patterns and microbial community structure, may promote an environment enriched in TMA-producing bacteria (e.g., some *Enterobacteriaceae, Prevotella* species) (Koeth et al., 2013; Sanchez-Rodriguez et al., 2020). Elevated TMAO levels are a strong, independent risk factor for atherosclerosis, thrombosis, and major adverse cardiovascular events (Cuevas-Sierra et al., 2019). TMAO promotes these effects by enhancing macrophage foam cell formation, stimulating platelet hyperreactivity, and promoting vascular inflammation (Zhu et al., 2016). Thus, CD can exacerbate a microbial metabolic pathway that directly links diet to cardiovascular complications, a leading cause of mortality in metabolic syndrome (Daniel et al., 2021).

Table 2 provides a summary of the key microbial metabolites discussed, their rhythmic functions, and the consequences of their dysregulation due to CD, integrating insights from the references cited throughout this section.

**Table 2:** Key microbial metabolites: rhythmic functions and consequences of their dysregulation.

Metabolite	Primary microbial producers	Rhythmic function in host	Consequence of rhythm loss in CD	References
Butyrate	*Faecalibacterium prausnitzii, Roseburia* spp., *Eubacterium hallii, Anaerostipes* spp	Colonocyte energy; HDAC inhibition; Barrier integrity (tight junctions); GPR109a activation; Entrainment of peripheral clocks (liver)	Impaired gut barrier → metabolic endotoxemia and inflammation; Desynchronized hepatic gluconeogenesis; Reduced anti-inflammatory tone; Colonic epithelial dysfunction	Zietak et al. (2016); Fujisaka et al. (2018); Santisteban et al. (2017)
Propionate	*Bacteroidetes* (many spp.), *Dialister, Veillonella, Roseburia inulinivorans, Phascolarctobacterium*	Substrate for hepatic gluconeogenesis; GPR41/43 ligand → PYY/GLP-1 release (satiety and insulin secretion)	Blunted postprandial satiety response → potential hyperphagia; Impaired postprandial insulin secretion; Disrupted energy harvest signaling	Chambers et al. (2015); Han et al. (2021); Perry et al. (2016)
Secondary BAs (e.g., DCA, LCA)	*Clostridium scindens* cluster, *Clostridium hiranonis*	FXR/TGR5 ligands; Regulate BA synthesis (via FGF19), glucose/lipid metabolism, GLP-1 secretion, energy expenditure and thermogenesis	Uncoordinated metabolic gene expression; Dysregulated BA pool and feedback; Impaired glucose tolerance; Reduced thermogenesis; Potential cholestatic risk	Bishehsari et al. (2015); Wahlström et al. (2016); Canfora et al. (2015)
Indoles (AhR ligands, e.g., IPA)	*E. coli, Lactobacillus* spp., *Bifidobacterium* spp., *Peptostreptococcus*	AhR activation: IL-22 production, immune regulation, barrier maintenance, anti-inflammatory	Compromised mucosal immunity and barrier; Increased gut permeability and systemic inflammation; Loss of protective IL-22 signaling	Cheng et al. (2020); Natividad et al. (2018); Yano et al. (2015)
TMA (precursor to TMAO)	*Enterobacteriaceae, Prevotella, Anaerococcus, Clostridium* (some strains)	None (microbial waste product)	Increased flux to TMAO → elevated risk of atherosclerosis, thrombosis, and major adverse cardiac events	Cresci and Bawden (2015); Koeth et al. (2013); Sanchez-Rodriguez et al. (2020)

## 4 Phytochemicals: therapeutic avenues for restoring microbial metabolite rhythms and metabolic homeostasis

Dietary phytochemicals present a multi-pronged, complementary, and often synergistic approach to counteract the deleterious effects of CD on the gut microbiome and host metabolism (Depommier et al., 2019). Their actions can be strategically categorized into three overlapping therapeutic avenues: modulating the structure of the microbial ecology, fine-tuning the metabolic output of the microbiota, and directly coordinating host cellular responses to reinforce circadian-metabolic coupling (Dyar et al., 2018).

### 4.1 As microecological modulators: reshaping the rhythmic community structure

Many phytochemicals function as prebiotics (selectively utilized host microorganisms conferring a health benefit) or as selective antimicrobials, thereby promoting the expansion of bacterial taxa that contribute to rhythmic, beneficial metabolite production while suppressing potential pathobionts (Farzi et al., 2018; Schroeder, 2019).

#### 4.1.1 Polyphenols

This vast and diverse class of compounds (flavonoids, stilbenes, phenolic acids, lignans) is often poorly absorbed in the upper gastrointestinal tract. As a result, a significant fraction reaches the colon intact, where they exert potent prebiotic effects and serve as substrates for microbial transformation (Ferrocino et al., 2015). Different polyphenol structures exhibit distinct selectivity for microbial groups. For example, resveratrol (a stilbene from grapes, blueberries, peanuts) has been consistently shown in rodent models of diet-induced obesity to increase the abundance of *Akkermansia muciniphila*. This mucin-degrading bacterium is strongly associated with improved gut barrier function. It also links to reduced metabolic endotoxemia and enhanced metabolic health. In addition, resveratrol also promotes the genarate of *Lactobacillus* and *Bifidobacterium* species (Bäckhed et al., 2004; Zhao et al., 2017). Anthocyanins (pigments in berries, red cabbage and black rice) and flavan-3-ols (abundant in green tea, cocoa, and apples) selectively stimulate the growth of *Bifidobacterium* and *Lactobacillus*, while often inhibit the proliferation of potential pathogenic bacteria such as *Clostridium perfringens* and *Bacteroides* spp. (Cardona et al., 2013). Ellagitannins (found in pomegranate, walnuts, strawberries) are not absorbed but are hydrolyzed and metabolized by specific gut bacteria (e.g., *Gordonibacter* spp.) to form urolithins (Gerhardt and Mohajeri, 2018). Urolithins themselves have anti-inflammatory and anti-aging properties and can further shape the microbial community toward a more favorable composition. Polyphenols can foster the growth of some beneficial SCFA producers (e.g., *Faecalibacterium prausnitzii, Roseburia* spp., *Eubacterium* spp.) and other beneficial symbionts, thereby shifting the compositional landscape of the gut microbiome (Li et al., 2020; Sanna et al., 2019). However, it is important to recognize that most studies demonstrating such compositional shifts have relied on single-timepoint fecal sampling, which cannot capture diurnal oscillatory patterns. Whether these compositional changes translate into restored rhythmicity of microbial metabolic output—i.e., whether phytochemicals reset the amplitude or phase of the microbial clock—remains largely unexamined. Current evidence suggests that polyphenols primarily create a more favorable ecological niche, which may enable the re-establishment of microbial rhythms when combined with appropriate temporal cues such as time-restricted feeding. Direct evidence of phytochemical-mediated rhythmic restoration requires future studies employing time-series metatranscriptomics and controlled feeding paradigms.

#### 4.1.2 Complex dietary matrices and synergistic effects

Whole or minimally processed plant foods (whole grains, legumes, nuts, seeds, and a diverse array of vegetables and fruits) provide synergistic packages of fermentable fibers, polyphenols, vitamins, and minerals. This combination supports a more diverse and functionally resilient microbial network (Gilbert et al., 2018). Microbial diversity is a key predictor of ecosystem stability and functional redundancy. A diverse microbiome is better equipped to resist stressors like CD. This resilience helps maintain critical functions, such as the rhythmic production of metabolites, even under challenging conditions (Le Chatelier et al., 2013; Sonnenburg and Bäckhed, 2016). The fiber provides the bulk substrate for growth, while polyphenols can selectively modulate the sub-populations within that growing community (Gupta et al., 2020).

#### 4.1.3 Critical considerations: distinguishing compositional modulation from rhythmic restoration

A critical caveat must be acknowledged when interpreting the microecological effects of phytochemicals. Current evidence predominantly demonstrates that these compounds increase the absolute abundance or relative proportion of beneficial taxa (e.g., *Akkermansia muciniphila, Bifidobacterium, Lactobacillus*) rather than directly restoring their diurnal oscillatory patterns. Whether phytochemicals reset the amplitude or phase of microbial rhythms, or merely provide a constant substrate supply that passively enriches certain populations, remains largely unexplored. Most studies rely on single-timepoint fecal sampling, which obscures dynamic rhythmicity and cannot distinguish between sustained compositional shifts and restored oscillatory function. Thus, while phytochemicals likely create a permissive ecological environment that facilitates rhythmic recovery—particularly when combined with temporal cues such as time-restricted feeding—their ability to directly entrain microbial clocks requires further investigation using time-series metatranscriptomics, longitudinal metabolomics, and controlled feeding paradigms.

### 4.2 As metabolic pathway modulators: calibrating microbial metabolic output

In addition to influencing microbial composition, specific phytochemicals can act like precise switches, directly regulating the enzymatic activity within gut bacteria. This allows them to adjust both the amount and type of key metabolites produced, effectively steering microbial metabolism toward a more beneficial output (He and Shi, 2017).

#### 4.2.1 Targeted regulation of bacterial enzyme activity

Berberine, an isoquinoline alkaloid derived from medicinal herbs like *Coptis chinensis*, has a well-demonstrated ability to inhibit microbial TMA lyases (CutC/D), the enzymes responsible for converting dietary choline and carnitine into trimethylamine (TMA). By reducing TMA production, berberine lowers subsequent host liver-mediated TMAO formation, offering a direct pharmacologic intervention in a CD-exacerbated cardiovascular risk pathway (Shi et al., 2018; Zhang et al., 2015a,b). Dietary Fibers [e.g., inulin, fructo-oligosaccharides (FOS), beta-glucan, arabinoxylan, pectin] are the primary fermentable substrates for SCFA production (Sun et al., 2019). Their consistent and appropriately timed intake ensures a steady supply of precursors (e.g., acetate for butyrogenesis) necessary for rhythmic SCFA synthesis. Different fibers have varying fermentation rates and selectively stimulate different bacterial groups, allowing for nuanced modulation of the SCFA profile (McNabney and Henagan, 2017; Zhao et al., 2018). Certain polyphenol-derived metabolites (e.g., urolithins, equol) may influence the activity of bacterial enzymes like bile salt hydrolase (BSH), which deconjugates BAs, or 7α-dehydroxylase, which forms secondary BAs. By subtly modulating these activities, phytochemicals can shift the bile acids pool composition toward a more favorable profile, potentially enhancing FXR/TGR5 signaling efficacy (Koppel et al., 2017).

#### 4.2.2 Strategic intervention in tryptophan metabolism pathways

Diets rich in cruciferous vegetables (broccoli, kale, and Brussels sprouts) provide glucosinolates, which are hydrolyzed to bioactive isothiocyanates like sulforaphane. These compounds, or their downstream metabolites, can directly activate the AhR, potentially compensating for deficits in microbially derived AhR ligands caused by dysbiosis (Natividad et al., 2018). Furthermore, phytochemicals can steer microbial tryptophan metabolism away from producing potentially harmful metabolites, such as indoxyl sulfate. Instead, they promote the generation of beneficial AhR agonists like indole-3-propionic acid (IPA), which possesses antioxidant and neuroprotective properties and is linked to renal and cardiovascular health benefits (Heiss and Olofsson, 2019).

### 4.3 As coordinators of host-microbe dialogue: reinforcing host circadian and metabolic resilience

Phytochemicals also exert direct effects on host cellular pathways that are integral to both circadian timing and metabolic sensing, thereby improving the host's intrinsic ability to interpret and respond appropriately to microbial signals, even under conditions of CD (Holmes et al., 2017).

#### 4.3.1 Activation of key host metabolic and circadian regulatory nodes

AMPK Pathway Activation: several polyphenols, including resveratrol and quercetin, are potent activators of AMP-activated protein kinase (AMPK), the central cellular energy sensor. AMPK activation promotes catabolic processes (fatty acid oxidation, glucose uptake) and inhibits anabolic ones (lipogenesis, protein synthesis). Intriguingly, AMPK is not just a metabolic sensor but also a circadian regulator. It phosphorylates and destabilizes the core clock protein CRY1, thereby influencing circadian period length (Lamia et al., 2009) and linking cellular energy status directly to the molecular clockwork.

SIRT1 Pathway Activation: resveratrol is a well-known activator of sirtuin 1 (SIRT1), a NAD+-dependent deacetylase. SIRT1 deacetylates both histones and specific proteins, including core clock components like BMAL1 and PER2. This activity is crucial for maintaining robust circadian amplitude, mitochondrial biogenesis, and metabolic efficiency. Its activation can reinforce circadian rhythmicity, improve insulin sensitivity, and enhance oxidative metabolism (Chang and Guarente, 2013).

Nrf2 Pathway Activation: curcumin (from turmeric) and sulforaphane (from broccoli sprouts) are well-characterized natural activators of nuclear factor erythroid 2–related factor 2 (Nrf2), the master transcriptional regulator of the cellular antioxidant and anti-inflammatory response. Oxidative stress is a known disruptor of circadian clocks. By potently inducing a battery of cytoprotective genes (e.g., heme oxygenase-1, NAD(P)H quinone dehydrogenase 1), Nrf2 activation helps maintain cellular redox balance, protects against clock protein damage, and supports metabolic health in the face of inflammatory stressors (Zhang et al., 2015a,b).

By bolstering these interconnected AMPK-SIRT1-Nrf2 pathways, phytochemicals enhance metabolic flexibility (the ability to switch between fuel sources) and strengthen the host's internal circadian framework. This makes peripheral tissues more resilient and more appropriately responsive to the rhythmic signals from a recovering microbiome (Johnston et al., 2016).

#### 4.3.2 Direct reinforcement of the intestinal barrier

Chronic CD and associated dysbiosis are key drivers of increased intestinal permeability (“leaky gut”) (Kang et al., 2017). Curcumin and quercetin possess strong anti-inflammatory (inhibiting NF-κB signaling) and antioxidant properties that have been shown to stabilize tight junction proteins and reduce intestinal permeability in animal models (Cani et al., 2008). By strengthening the gut barrier, these compounds limit the entry of pro-inflammatory bacterial products (e.g., LPS) into circulation. This action alleviates chronic low-grade inflammation (metabolic endotoxemia)—a key driver that connects gut dysbiosis to systemic insulin resistance and metabolic disease (Kashi et al., 2016).

### 4.4 The chrono-therapeutic potential: optimizing the timing of phytochemical intake

The emerging principle of chronotherapy aims to optimize treatment timing by aligning it with the body's natural rhythms, thereby maximizing efficacy and minimizing side effects (Moskalev and Vaiserman, 2017). This approach holds immense promise for guiding the application of phytochemicals. Their beneficial effects may be significantly amplified if administered at a time that synergizes with both host and microbial physiology (Korpela, 2018).

#### 4.4.1 Rationale for optimizing intake timing

The concept of chrono-nutrition extends to phytochemicals. Administering prebiotic fibers or polyphenol-rich foods during the early active phase/feeding window could maximize SCFA production to coincide with the host's peak insulin sensitivity and heightened energy demand. This aligns microbial metabolic support with host metabolic need (Larsen et al., 2010; Li et al., 2017). Conversely, certain compounds with potent antioxidant or tissue-repair properties (e.g., some polyphenols) might be more beneficial when taken in the evening to support overnight recovery and repair processes, which are under circadian control (Li et al., 2018). Preliminary evidence supports this principle. For instance, nobiletin—a citrus flavonoid that enhances circadian rhythm by activating specific clock proteins (RORα/γ)—has shown time-dependent effects in mice. When administered in sync with the biological clock, it more effectively improves metabolic health in high-fat diet models (He et al., 2016).

#### 4.4.2 Synergistic integration with time-restricted eating (TRE)

Combining targeted phytochemical supplementation with TRE represents a powerful, synergistic lifestyle and dietary strategy (Lin et al., 2012). TRE itself is a potent intervention that provides a strong, consistent external cue, powerfully entraining both host peripheral clocks and microbial community rhythms (Panda, 2016). Phytochemicals can function within this temporal framework in two key ways. Acting like precision tools, they can selectively promote the growth of beneficial microbes favored by TRE. Concurrently, they provide direct support to host circadian-metabolic pathways during fasting, thereby enhancing autophagy and stress resistance (Liu et al., 2017). Preliminary animal studies suggested that the metabolic benefits of certain polyphenols were indeed more pronounced when their consumption ws contained within a constrained daily feeding window, highlighting the importance of timing (Lozupone et al., 2012).

Table 3 outlines examples of key phytochemicals, their proposed mechanisms of action on the microbiome and host, and the rationale for their potential optimal timing, drawing upon the evidence reviewed in this section.

**Table 3:** Examples of phytochemicals, their microbial and host targets, and potential chrono-therapeutic value.

Phytochemical class/Example	Dietary source	Proposed microbiome/ Metabolite effects	Key host targets/Pathways	Potential optimal timing rationale	References
Resveratrol (Stilbene)	Grapes, blueberries, peanuts, red wine	↑*Akkermansia muciniphila, Lactobacillus*; ↑SCFA production; Modifies BAs pool composition	Activates SIRT1, AMPK; Anti-inflammatory (NF-κB inhibition); Improves mitochondrial function	Morning/early active phase to synergize with SIRT1/AMPK-driven metabolic activation and daytime energy metabolism	Bäckhed et al. (2004); Zhao et al. (2017); Cardona et al. (2013)
Curcumin (Curcuminoid)	Turmeric root	↑*Bifidobacterium, Lactobacillus*; ↑butyrate production; ↓LPS-producing bacteria; may modulate BA metabolism	Potent Nrf2 activator; Inhibits NF-κB; Strengthens tight junctions; Anti-inflammatory and antioxidant	With main meals to enhance bioavailability via piperine/fats and to directly counteract meal-induced oxidative stress and inflammation	Zhang et al. (2015a,b); Kashi et al. (2016)
Quercetin (Flavonol)	Onions, apples, capers, buckwheat, berries	Modulates microbial composition (↑beneficials); enhances gut barrier integrity; may act as prebiotic	Antioxidant; Anti-inflammatory; stabilizes mast cells	Morning/afternoon to bolster daytime antioxidant defenses and support metabolic sensing during active period	Cani et al. (2008); Lamia et al. (2009)
Dietary fiber (e.g., Inulin, FOS)	Chicory root, garlic, asparagus, Jerusalem artichoke, oats	↑Abundance of SCFA producers (*Bifidobacterium, Roseburia, Faecalibacterium*); Primary fermentable substrate for SCFA generation	SCFA receptors (GPR41/43, Olfr78); HDAC inhibition; ileal brake via PYY/GLP-1	Early in the feeding window (e.g., breakfast) to provide substrate for robust daytime SCFA production, aligning microbial energy harvest with host energy needs	Sun et al. (2019); McNabney and Henagan (2017); Zhao et al. (2018)
Sulforaphane (Isothiocyanate)	Broccoli sprouts, kale, cabbage, Brussels sprouts	May influence microbial tryptophan metabolism/AhR ligand production; antimicrobial properties	Potent Nrf2 activator; Induces phase II detoxification enzymes; AhR agonist	With meals (especially from raw/sprouted sources) to coincide with metabolic activation of the Nrf2 pathway and nutrient metabolism	Heiss and Olofsson (2019); Zhang et al. (2015a,b)
Berberine (Isoquinoline Alkaloid)	*Coptis chinensis* (Goldthread), barberry, Oregon grape	Inhibits TMA-producing bacteria; modulates BA metabolism; may have broad antimicrobial effects	Activates AMPK; improves insulin sensitivity; modulates lipid metabolism; may activate SIRT1	Timing less clear; often aligned with meals in clinical practice to mitigate postprandial hyperglycemia and metabolic disturbance. May benefit from chrono-optimization studies	Sun et al. (2019); Koppel et al. (2017)
Nobiletin (Polymethoxy-flavone)	Citrus fruit peels (oranges, tangerines)	Understudied, but may influence microbiome via systemic effects	RORα/γ agonist; potent clock amplitude enhancer; improves circadian rhythm and metabolic parameters	Evening administration has shown benefit in rodent models, potentially to reinforce the ascending phase of clock gene expression and overnight metabolic repair	He et al. (2016)

## 5 Translational challenges and future directions

### 5.1 Challenges in translating mechanistic insights to clinical practice

Significant translational hurdles must be overcome to convert these concepts into effective and personalized therapies, despite the elegant mechanistic models from preclinical studies and the growing body of supportive human data (Mardinoglu and Nielsen, 2012).

#### 5.1.1 Profound complexity and inter-individual variability in human studies

The human gut microbiome exhibits immense variability between individuals. This diversity is shaped by a lifetime of unique exposures—including long-term diet, host genetics, geographic and cultural background, medication history (particularly antibiotics), mode of birth, and the early-life environment (Meijnikman et al., 2018). This results in highly personalized baseline microbial communities and consequently, highly variable responses to both circadian disruptors and phytochemical interventions (Zeevi et al., 2015; Zmora et al., 2018). A universal “one-size-fits-all” prescription for phytochemical type, dose, and timing is unlikely to yield consistent results across a heterogeneous population (Miro-Blanch and Yanes, 2019). Furthermore, achieving and objectively monitoring long-term adherence to precise dietary timing regimens or specific phytochemical supplementation schedules in free-living individuals presents a major practical and behavioral challenge (Monda et al., 2017).

#### 5.1.2 Establishing direct causality and defining clinically useful biomarkers

While rodent FMT studies are powerful for establishing causality in controlled settings, human evidence remains largely correlational. To definitively prove that restoring a specific microbial metabolite rhythm directly improves a clinical metabolic endpoint in humans requires complex, expensive, and logistically demanding longitudinal intervention studies (Nagpal et al., 2018). These studies need to incorporate high-frequency sampling (multiple samples over 24-h periods) to capture dynamic changes in microbial composition, metabolite fluxes, and host physiology (O'Keefe, 2016). There is a pressing need to identify and validate robust, non-invasive biomarkers of a “healthy, rhythmic microbiome” and of circadian alignment (Parada Venegas et al., 2019). Such biomarkers (e.g., specific metabolite ratios in urine or blood collected at strategic times, microbial gene expression signatures from serial stool samples) could serve as intermediate endpoints in clinical trials, accelerating the development of chrono-nutritional therapies.

#### 5.1.3 Pharmacokinetics, bioavailability, and food matrix effects

The bioavailability of many polyphenols is inherently low and highly variable. Key influencing factors include: the food source (whole food vs. extract), culinary processing, and the co-consumption of other foods (e.g., fats which can enhance absorption). Additionally, an individual's gut microbiota composition plays a crucial role, as it transforms many polyphenols into their active forms (Parker et al., 2020). This adds another formidable layer of complexity to standardizing dosing and predicting individual clinical responses. Personalized approaches may need to account for an individual's “metabotype”—their capacity to metabolize specific phytochemicals (Patnode et al., 2019; Petrosino, 2018).

#### 5.1.4 Species differences in circadian physiology and translational limitations

A critical translational challenge arises from the predominant use of nocturnal rodents (mice, rats) in mechanistic studies. Rodents are active during the dark phase and consume the majority of their calories during this period, which is the opposite of diurnal humans. Consequently, concepts such as “time-restricted feeding” or “night-time eating” carry opposite connotations: in rodents, feeding during the light (rest) phase is considered “mistimed,” whereas in humans, feeding during the dark phase is aberrant. Moreover, rodents have significantly higher basal metabolic rates and shorter circadian periods, which may influence the magnitude and kinetics of microbial and metabolic responses to feeding interventions. While TRF has shown robust benefits in both rodents and humans, the direct translation of feeding time windows (e.g., 8- vs. 12-h) and the underlying mechanisms may differ. Future studies should prioritize human trials with rigorous circadian phenotyping and, when using animal models, consider diurnal species (e.g., zebrafish or non-human primates) to better approximate human circadian physiology.

### 5.2 Future research directions: toward precision chrono-nutrition

To bridge the gap between mechanistic insight and meaningful clinical application, future research must prioritize the following interconnected strands of investigation.

#### 5.2.1 Multi-timepoint, longitudinal multi-omics profiling in humans

The field must move decisively beyond single-timepoint, static “snapshots” of the microbiome. The future lies in longitudinal “time-series” studies that track the same individuals over time, under different conditions (e.g., during shift work schedules, before and after TRE or phytochemical interventions) (Jiao et al., 2018). By integrating multi-omics data—metagenomics (microbial identity), metatranscriptomics (gene activity), metabolomics (from feces, serum, and urine), and host transcriptomics/epigenomics—across multiple 24-h cycles in well-phenotyped cohorts, we can construct detailed, personalized “metabolic rhythm maps.” This systems biology approach can identify which specific nodes in the host-microbe-metabolite network are most disrupted in a given individual or population and predict personalized intervention targets (Thaiss et al., 2014; Dyar et al., 2018).

#### 5.2.2 Developing the framework for personalized chrono-nutrition

The ultimate goal is to tailor dietary and phytochemical interventions to an individual's unique physiological profile. Achieving this requires converging several key data streams (Ridaura et al., 2013). First, assessing chronotype through questionnaires (e.g., Munich Chronotype Questionnaire) or objective measures like dim-light melatonin onset. Second, performing deep profiling of the gut microbiome and metabolome to understand baseline community structure and metabolic rhythms. Finally, implementing dynamic physiological monitoring using devices like continuous glucose monitors and activity trackers to capture real-time circadian and metabolic data. In addition, machine learning algorithms can then integrate this multimodal data to generate personalized, real-time adaptive recommendations that evolve with changes in an individual's physiology and behavior. These insights would guide personalized recommendations, not only for the optimal timing of meals and specific phytochemical intake, but also for selecting the types of fibers or polyphenols best suited to nourish the individual's unique microbial ecosystem and align with their circadian profile (Zeevi et al., 2015; Bashiardes et al., 2018; Kolodziejczyk et al., 2019).

#### 5.2.3 Exploring next-generation microbiome-targeted therapeutics

Future therapeutic strategies are poised to move beyond conventional approaches in several key directions. Firstly, engineered live biotherapeutic products could be designed as active agents—for instance, bacteria programmed to produce beneficial metabolites like butyrate in response to specific host or dietary cues (Park and Im, 2020). Secondly, the direct use of purified microbial metabolites (e.g., stabilized butyrate derivatives) as “signal restoration therapies” offers a precise way to replenish rhythmic host signals, though it may lack the ecological benefits of modulating the microbiome itself (De Vadder et al., 2014; Plovier et al., 2017). Finally, there is a pressing need for multimodal chrono-therapy trials that systematically test combinations (e.g., time-restricted eating + specific polyphenols + timed light exposure) to reset the clock-microbiome axis and improve metabolic outcomes in targeted populations (Sen et al., 2021).

## 6 Conclusion

In conclusion, circadian disruption has emerged as a potent driver of metabolic disease, acting in large part through desynchronization of the gut microbiome and its rhythmic metabolite output. Dietary phytochemicals offer a unique multi-targeted strategy to restore this axis by remodeling microbial ecology, calibrating metabolic output, and reinforcing host circadian resilience. However, critical questions remain: Do phytochemicals directly reset microbial rhythms or merely create a permissive environment? How do species differences in circadian physiology affect translation from rodents to humans? Addressing these gaps will require time-series multi-omics and carefully designed human chrono-nutrition trials. By integrating phytochemicals into a personalized chrono-nutrition framework, we can move toward effective interventions that harness the clock-microbiome-metabolite axis to combat metabolic disease.
